# Pneumocystis jirovecii Pneumonia in an Adolescent With Acute Lymphoblastic Leukemia Due to Non-adherence to Prophylaxis: A Case Report

**DOI:** 10.7759/cureus.117083

**Published:** 2026-09-28

**Authors:** Petra Pelcic, Lucija Ruzman

**Affiliations:** 1 Department of Pediatrics, Division of Hematology and Oncology, Clinical Hospital Center Rijeka, Rijeka, HRV; 2 Department of Pediatrics, Faculty of Medicine, University of Rijeka, Rijeka, HRV

**Keywords:** acute lymphoblastic leukemia, adolescent, medication non-adherence, pneumocystis jirovecii pneumonia, trimethoprim-sulfamethoxazole

## Abstract

*Pneumocystis jirovecii* pneumonia (PJP) is a severe and potentially life-threatening opportunistic infection in immunocompromised patients, especially those with hematologic malignancies. Although routine prophylaxis with trimethoprim-sulfamethoxazole (TMP-SMX) has significantly reduced its incidence, breakthrough infections still occur, most commonly due to poor adherence to prophylactic therapy. This case report highlights the risks of non-adherence to PJP prophylaxis in adolescents and underscores the importance of early diagnostic suspicion and multidisciplinary management. A 15-year-old female patient with acute lymphoblastic leukemia presented during her seventh month of maintenance therapy (methotrexate and 6-mercaptopurine) in a compromised general condition with tachycardia, tachypnea, and hypoxemia, following a 24-hour history of fever and dry cough. Heteroanamnestic data revealed prolonged non-adherence to TMP-SMX prophylaxis. Imaging showed diffuse bilateral interstitial infiltrates on chest radiography and ground-glass opacities on high-resolution computed tomography (HRCT). PJP was confirmed via polymerase chain reaction (PCR) from bronchoalveolar lavage and a positive serum 1,3-β-D-glucan test. Treatment included intravenous TMP-SMX, corticosteroids, and high-flow nasal cannula oxygen therapy. The course was complicated by pancytopenia, likely secondary to TMP-SMX, and febrile neutropenia. A multidisciplinary approach, including social and psychiatric support, addressed non-adherence. The patient was discharged in good condition; maintenance therapy was continued, and prophylaxis was switched to monthly aerosolized pentamidine administered in the hospital setting. PJP prevention in pediatric oncology remains a significant challenge, as inconsistent adherence can rapidly lead to severe clinical deterioration. Our experience suggests that successful outcomes depend on addressing psychosocial barriers early. Transitioning to supervised, hospital-administered alternatives, such as aerosolized pentamidine, alongside multidisciplinary support, may help ensure adherence to prophylaxis in vulnerable oncology patients.

## Introduction

Acute lymphoblastic leukemia (ALL) is the most common malignancy in children. Globally, leukemia accounts for approximately 37% of all newly diagnosed childhood cancers, with ALL representing nearly two-thirds of these cases. This is the most common pediatric malignancy, with a peak incidence between one and four years of age [1]. Over the past two decades, remarkable advances in the treatment of ALL have resulted in five-year survival rates exceeding 90% in many developed countries [2].

Despite these advances, intensive chemotherapy and prolonged immunosuppression significantly increase the risk of opportunistic infections. Among these, *Pneumocystis jirovecii *pneumonia (PJP) remains one of the most severe and potentially life-threatening conditions. It is caused by the opportunistic fungus *Pneumocystis jirovecii *(formerly known as *Pneumocystis carinii*). Before the introduction of routine prophylaxis, PJP was a leading cause of infectious mortality in pediatric patients with ALL. The use of trimethoprim-sulfamethoxazole (TMP-SMX) has markedly reduced its incidence and associated morbidity and mortality.

However, despite well-established prophylactic strategies, PJP continues to occur in patients with profound T-cell deficiency or dysfunction, including those receiving corticosteroid therapy or myeloablative conditioning for hematopoietic cell transplantation, those with high-grade graft-versus-host disease or respiratory cytomegalovirus coinfection, and those with poor adherence to prophylaxis, particularly among adolescents [3].

This case report describes a severe PJP presentation due to inadequate adherence to prophylaxis, underscoring the importance of timely recognition, attention to psychosocial factors, and a multidisciplinary approach to managing immunocompromised oncology patients, particularly those from socially vulnerable backgrounds.

## Case presentation

A 15-year-old female patient with ALL presented to the hematology outpatient clinic for a routine follow-up visit in poor general condition, following a 24-hour history of high-grade fever and dry cough. On presentation, she was febrile (temperature: 38°C), tachycardic (heart rate: 150/min), tachypneic (respiratory rate: 40/min), dyspneic, and hypoxemic, with an SpO₂ of 85% on room air. The patient had been treated according to the ALL-IC BFM 2009 Protocol for the intermediate-risk group and had previously completed induction, early intensification, and consolidation therapy without significant treatment-related complications. At the time of evaluation, she had been receiving maintenance therapy for seven months, consisting of methotrexate and 6-mercaptopurine, along with chemoprophylaxis with TMP-SMX.

During the initial history-taking, the patient reported experiencing only mild symptoms consistent with a "common cold" and emphasized adherence to her prescribed oral PJP prophylaxis. Patients undergoing treatment at our institution are routinely instructed to seek immediate medical evaluation if they develop high-grade fever. Despite developing a fever of up to 39°C one day before presentation, she attended her scheduled follow-up visit rather than seeking urgent medical care. At presentation, signs of clinical deterioration were already evident. The patient was urgently admitted to the hematology-oncology department for close monitoring, with the intensive care team alerted for potential immediate transfer due to her severe respiratory distress. Within 1-2 hours during diagnostic workup, including laboratory testing and radiologic evaluation, she developed worsening tachypnea, with a respiratory rate of up to 50/min, progressive dyspnea, and a persistent dry, irritative cough. Further history-taking revealed that she had been independently administering her oral medications without caregiver supervision. As her respiratory status worsened, she became increasingly distressed and ultimately disclosed prolonged non-adherence to TMP-SMX prophylaxis. This was further supported by the caregivers' discovery of two unopened boxes of TMP-SMX tablets that had not been used. Adherence to 6-mercaptopurine and methotrexate was assessed simultaneously. Both medications were reportedly taken as prescribed, which was also supported by the regular weekly complete blood counts showing persistent leukocyte counts between 2×10⁹/L and 3×10⁹/L during maintenance therapy.

The patient came from a socially vulnerable family environment characterized by limited caregiver support and financial constraints. She was primarily cared for by her elderly grandparents, while her mother resided abroad and had limited involvement in her daily care; her father was incarcerated. Before the diagnosis of ALL, the patient had demonstrated behavioral difficulties, which improved during intensive treatment with the support of the multidisciplinary team, including a psychologist and social worker. Following the transition to maintenance therapy, opportunities for close psychosocial monitoring became more limited, and the healthcare team was not aware that, despite being a minor, the patient had assumed responsibility for independently managing and taking her oral medications without caregiver supervision.

Based on the clinical presentation, the differential diagnosis included bacterial and viral pneumonia, invasive fungal infection, particularly aspergillosis, and drug-induced pneumonitis. Given the rapid clinical deterioration and the eventual disclosure of prolonged non-adherence to PJP prophylaxis, PJP was strongly suspected, and empiric intravenous TMP-SMX was initiated at 20 mg/kg/day of the trimethoprim component, administered in four divided doses for 21 days. Adjunctive corticosteroid therapy with oral prednisone was initiated at 40 mg twice daily for five days, followed by 40 mg once daily for five days and then 20 mg once daily for 11 days, for a total duration of 21 days. Respiratory support with high-flow nasal cannula (HFNC) was required during the first three days of hospitalization, after which it was successfully discontinued. Initial laboratory evaluation showed leukopenia, mild anemia, normal platelet count, and markedly elevated C-reactive protein (Table 1).

**Table 1:** Laboratory evaluation at admission

Laboratory parameter	Patient value	Reference range
White blood cell	1.1×10⁹/L	4-11×10⁹/L
Hemoglobin	10.9 g/dL	13.5-17.5 g/dL
Platelets	182×10⁹/L	150-450×10⁹/L
Erythrocyte sedimentation rate	88 mm/hr	0-15 mm/hr
C-reactive protein	210 mg/L	0-5 mg/L
Procalcitonin	0.44 µg/L	<0.046 µg/L

Chest radiography demonstrated diffuse bilateral interstitial infiltrates (Figure 1).

**Figure 1:**
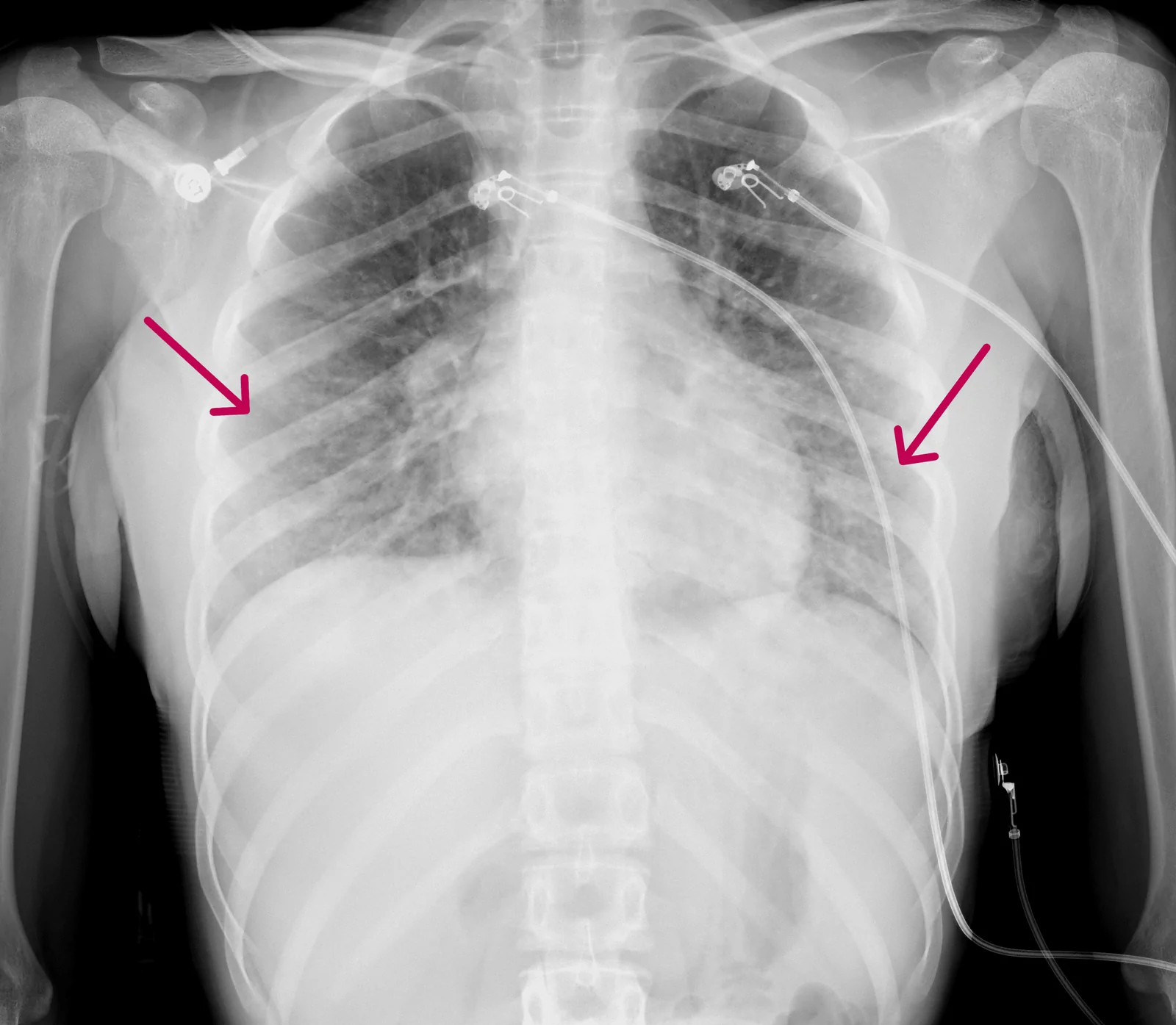
Chest X-rays obtained on the first day of hospitalization showing bilateral interstitial infiltrates, more prominent in the lower zones (arrows)

High-resolution computed tomography (HRCT) of the chest revealed diffuse bilateral ground-glass opacities (Figure 2). 

**Figure 2:**
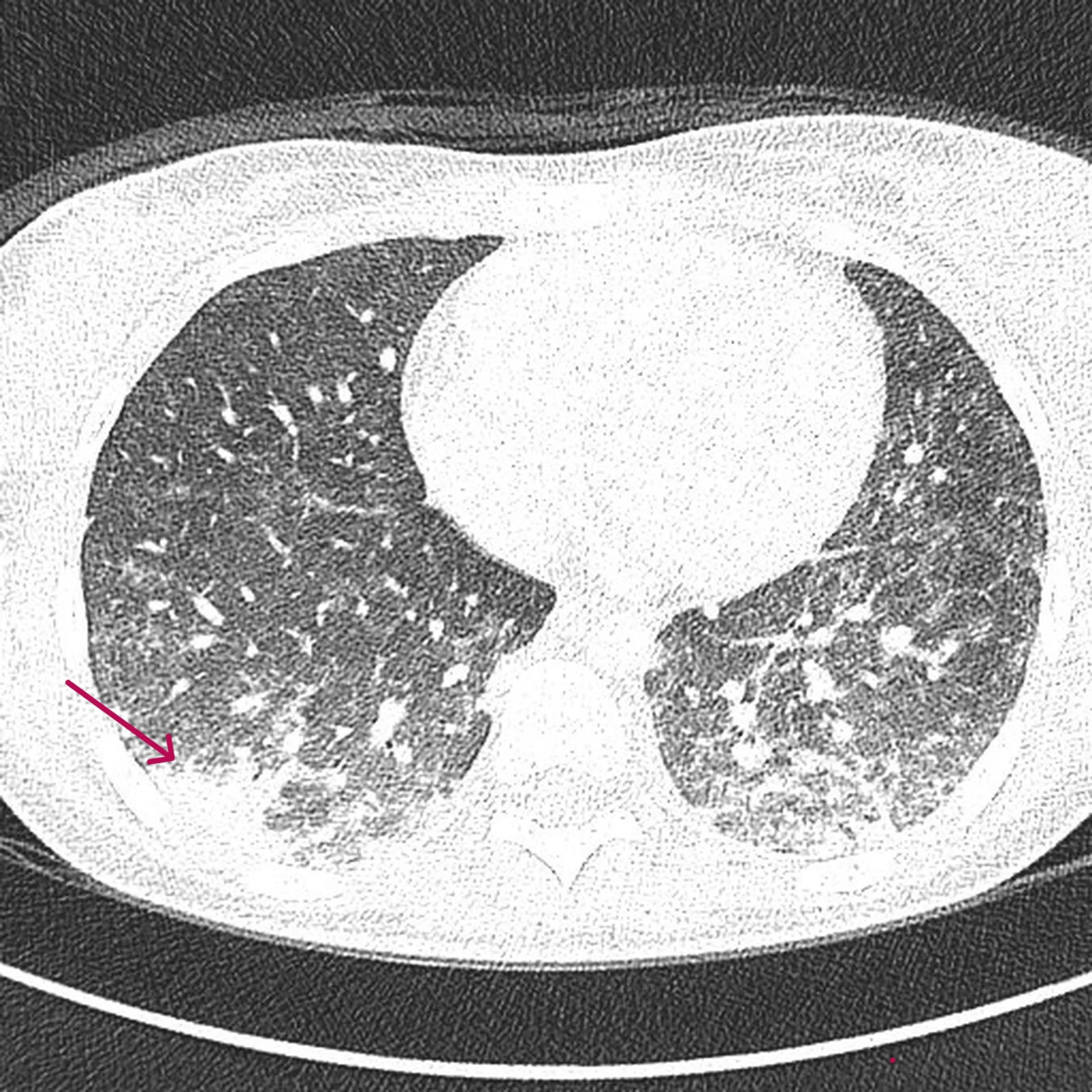
Chest computed tomography performed on the second day of hospitalization demonstrating extensive bilateral lower lobe ground-glass opacities with areas of posterior segment consolidation (arrow)

Bronchoscopy revealed mild mucosal erythema of the right main and intermediate bronchi, without pathological secretions. Bronchoalveolar lavage (BAL) was performed, and polymerase chain reaction (PCR) analysis of the BAL specimen confirmed *Pneumocystis jirovecii *infection. Additionally, serum 1,3-β-D-glucan was positive.

The patient's hospital course was complicated by severe pancytopenia, with an absolute neutrophil count of 0.29×10⁹/L, hemoglobin of 76 g/L, and a platelet count of 16×10⁹/L. This bone marrow suppression was considered most likely related to high-dose and prolonged intravenous TMP-SMX therapy, leading to a temporary interruption of maintenance chemotherapy. She subsequently developed febrile neutropenia and was treated with empiric cefepime according to institutional guidelines. Granulocyte colony-stimulating factor (G-CSF) was administered until recovery of myelopoiesis. After clinical improvement and hematologic recovery, the patient was discharged in good general condition, and maintenance chemotherapy was subsequently resumed.

During hospitalization for PJP, the patient and her family received social, psychological, and psychiatric support. Due to concerns regarding treatment adherence and patient safety, caregiving arrangements were formally reassessed. A social welfare assessment was conducted, and several multidisciplinary meetings were held. Ultimately, it was determined that continued care by the grandparents, with increased supervision arranged by social services, was in the patient's best interest.

To ensure adherence, PJP prophylaxis was changed to monthly aerosolized pentamidine administered in the hospital setting. The patient completed maintenance chemotherapy, and prophylaxis was continued for three months after the completion of chemotherapy. She remains in remission from ALL and attends regular follow-up appointments accompanied by her grandparents and a supervisor assigned by social services. The hospital team supported her transition to a more suitable educational setting; however, she ultimately discontinued secondary education.

## Discussion

PJP remains a severe opportunistic infection and a life-threatening complication in pediatric ALL. Although the efficacy of TMP-SMX prophylaxis is well established, its effectiveness in clinical practice largely depends on consistent adherence, particularly during the maintenance phase, when treatment is primarily managed in the outpatient setting. In our patient, the development of PJP during maintenance therapy illustrates the disparity between guideline-recommended prophylaxis and real-world adherence challenges, particularly in the adolescent population.

The clinical presentation of PJP is often nonspecific, typically including progressive dyspnea, fever, nonproductive cough, and hypoxemia, which may delay diagnosis in immunocompromised patients. In the present case, the rapid onset of respiratory symptoms, together with heteroanamnestic data suggestive of poor adherence to prophylaxis and characteristic radiological findings, prompted early diagnostic evaluation and initiation of targeted therapy [4-6].

A definitive PJP diagnosis relies on identifying the organism in respiratory specimens; BAL is the gold standard due to its higher sensitivity than induced sputum, particularly in the pediatric population. PCR assays further enhance diagnostic sensitivity, although their specificity may be limited in immunocompromised hosts. Additionally, serum 1,3-β-D-glucan supports the diagnosis; however, given its lack of specificity, its primary clinical value lies in its high negative predictive value, making it useful for excluding the disease [6,7].

High-dose intravenous TMP-SMX remains the first-line therapy for PJP, but its use is often limited by significant bone marrow suppression. In this patient, management was complicated by severe pancytopenia, likely resulting from the additive myelosuppressive effects of high-dose TMP-SMX and ongoing maintenance chemotherapy. This case illustrates the clinical challenge of balancing effective antimicrobial treatment with the risk of hematologic toxicity in oncology patients [5,7].

TMP-SMX remains the first-line agent for PJP prophylaxis due to its superior efficacy and cost-effectiveness [8]. However, its reliance on consistent oral administration may limit its effectiveness in patients with significant adherence challenges. While atovaquone and dapsone are established second-line alternatives, aerosolized pentamidine offers a practical option in select cases, particularly when supervised administration is feasible. Clinical data support the use of aerosolized pentamidine as an effective prophylactic strategy when TMP-SMX cannot be reliably administered [4,9]. In one of the largest pediatric cohorts, Quinn et al. reported no cases of probable or proven PJP and only four breakthrough infections (0.5%) among 754 patients receiving pentamidine prophylaxis [9]. Despite these favorable outcomes, the comparative efficacy of second-line agents remains a concern. Geerlinks et al. demonstrated that children on alternative regimens, including aerosolized pentamidine, faced a significantly higher risk of breakthrough infections compared to those on TMP-SMX (OR: 0.15; p=0.02). This disparity underscores that while aerosolized pentamidine is effective, it remains clinically inferior to sulfonamides, and a TMP-SMX rechallenge should be considered whenever practical [10]. Additionally, aerosolized pentamidine requires monitoring for potential adverse effects, including cough, bronchospasm, and gastrointestinal symptoms [9]. Table 2 summarizes the main pharmacologic options for PJP prophylaxis in pediatric patients, adapted from Cross et al. and Children's Oncology Group supportive care recommendations [4,8]. 

**Table 2:** Pharmacologic options for PJP prophylaxis in pediatric patients PJP:* Pneumocystis jirovecii *pneumonia; TMP-SMX: trimethoprim-sulfamethoxazole; G6PD: glucose-6-phosphate dehydrogenase

Agent	Route	Dosing and frequency	Advantages	Limitations
TMP-SMX	Oral	TMP 2.5 mg/kg/dose (75 mg/m²/dose), twice daily on three consecutive days per week (maximum 160 mg/dose)	First-line agent; highest efficacy; low cost; also provides protection against *Toxoplasma gondii*	Requires reliable oral adherence; may cause myelosuppression, rash, hepatotoxicity, or gastrointestinal adverse effects
Dapsone	Oral	2 mg/kg/day once daily (maximum 100 mg/day)	Oral alternative when TMP-SMX is not tolerated	Contraindicated in G6PD deficiency; risk of hemolysis and methemoglobinemia; requires adherence
Atovaquone	Oral	Age 1-3 months: 30 mg/kg/day once daily; age 4-24 months: 45 mg/kg/day once daily; age >24 months: 30 mg/kg/day once daily	Useful alternative in patients intolerant of TMP-SMX or dapsone	Expensive; requires daily oral intake and good absorption; should be taken with food; limited by adherence
Aerosolized pentamidine	Inhaled	300 mg once monthly (in children able to cooperate with inhalation, usually ≥5 years)	Supervised hospital administration possible; useful when oral adherence is unreliable	Less effective than TMP-SMX; no *Toxoplasma* coverage; may cause cough or bronchospasm; suitable only for cooperative children
Intravenous pentamidine	Intravenous	4 mg/kg/dose every 2-4 weeks	Option when oral or inhaled prophylaxis is not feasible; allows supervised administration	Requires hospital access; less commonly used; potential systemic adverse effects

Medication non-adherence in pediatric oncology is a complex, multifactorial challenge influenced by patient and caregiver characteristics, therapy-specific variables, disease-related factors, health system constraints, and broader social and economic determinants. Commonly reported factors associated with non-adherence include financial difficulties, patient age, behavioral characteristics, and caregiver-related factors [11]. A systematic review found that 21-60% of adolescents and young adults (AYAs) with cancer are non-adherent to oral medications [12], which can substantially compromise treatment efficacy, elevate the risk of infection or disease relapse, and adversely affect overall clinical outcomes. Improving adherence to oral medications in pediatric oncology generally necessitates a multifaceted approach. Education-based and psychosocial support strategies are most commonly employed. Additional interventions include medication diaries, peer counseling, reminder systems, and behavioral support tools, all of which aim to enhance treatment comprehension, establish consistent routines, and increase motivation among patients and caregivers [13].

The psychological burden associated with prolonged maintenance therapy in pediatric oncology has been described as a contributing factor to treatment non-adherence, particularly among adolescents. The transition from intensive, closely supervised treatment phases to the self-managed maintenance phase may negatively affect adherence. In adolescents, this shift is further influenced by developmental factors, including the drive for autonomy and the need for social integration, which may conflict with the demands of long-term oral therapy. Adherence in this setting is multifactorial and may also be influenced by family dynamics, treatment complexity, and individual risk perception [14,15]. The 2025 European Standards of Care for Children and Adolescents with Cancer emphasize the need to integrate psychosocial care throughout treatment and follow-up to support the emotional, psychological, social, and physical well-being of patients and their families [16]. An interdisciplinary approach to psychosocial care is essential, with social workers serving as a key link between the healthcare system, patients, families, and the broader community. However, implementation in clinical practice may remain challenging because of insufficiently defined institutional pathways, limited financial resources, inadequate institutional support, and insufficient staffing [17]. In such circumstances, supervised prophylactic strategies may be necessary when reliable adherence to oral medication cannot be ensured.

This case highlights that apparent failure of prophylaxis may, in some instances, reflect non-adherence rather than true pharmacological inefficacy. Early recognition of adherence-related risk factors, combined with a multidisciplinary approach and, when necessary, supervised prophylactic strategies, may be essential to ensure effective infection prevention in adolescent patients undergoing prolonged maintenance therapy.

## Conclusions

This case underscores the importance of clinicians maintaining awareness of patients' social context and consistently reassessing adherence to medical recommendations. This process involves actively re-evaluating, confirming, and questioning whether instructions are being followed, especially in situations where non-adherence may result in life-threatening complications. Clinicians should recognize that apparently minor or nonspecific clinical presentations can rapidly progress to severe disease, underscoring the need for ongoing evaluation of both patients and caregivers. Psychological support is necessary for patient empowerment and reintegration into daily life and the social environment. Additionally, when an inadequate social background is suspected, early involvement of social workers should be strongly considered, including their active engagement and, when appropriate, assessment of the patient's living environment, particularly when social circumstances may compromise patient safety and well-being.

## References

[1] Wang M, Bi Y, Fan Y, Fu X, Jin Y (2025). Global incidence of childhood cancer by subtype in 2022: a population-based registry study. EClinicalMedicine.

[2] Pui CH, Yang JJ, Hunger SP (2015). Childhood acute lymphoblastic leukemia: progress through collaboration. J Clin Oncol.

[3] Luo H, Wu P, Yang H (2025). Clinical analysis of Pneumocystis jirovecii pneumonia in children with malignant disease. BMC Pediatr.

[4] Cross SJ, Wolf J, Patel PA (2023). Prevention, diagnosis and management of Pneumocystis jirovecii infection in children with cancer or receiving hematopoietic cell therapy. Pediatr Infect Dis J.

[5] Dhingra D, Mandal A, Singh A (2018). Pneumocystis jirovecii pneumonia in children. J Pediatr Infect Dis.

[6] Weyant RB, Kabbani D, Doucette K, Lau C, Cervera C (2021). Pneumocystis jirovecii: a review with a focus on prevention and treatment. Expert Opin Pharmacother.

[7] Shankar SM, Nania JJ (2007). Management of Pneumocystis jiroveci pneumonia in children receiving chemotherapy. Paediatr Drugs.

[8] 8] Children's Oncology Group (2026). COG‑Endorsed Supportive Care Guidelines. https://childrensoncologygroup.org/collaborate/researchers/supportive-care-guidelines/.

[9] Quinn M, Fannin JT, Sciasci J (2018). Pentamidine for prophylaxis against Pneumocystis jirovecii pneumonia in pediatric oncology patients receiving immunosuppressive chemotherapy. Antimicrob Agents Chemother.

[10] Geerlinks AV, Campigotto A, Science M, Gupta S (2020). Impact of prophylaxis choice on risk of pneumocystis pneumonia in children with cancer: a case-control study. Eur J Cancer.

[11] Goh XT, Tan YB, Thirumoorthy T, Kwan YH (2017). A systematic review of factors that influence treatment adherence in paediatric oncology patients. J Clin Pharm Ther.

[12] McGrady ME, Pai AL (2019). A systematic review of rates, outcomes, and predictors of medication non-adherence among adolescents and young adults with cancer. J Adolesc Young Adult Oncol.

[13] Coyne KD, Trimble KA, Lloyd A (2019). Interventions to promote oral medication adherence in the pediatric chronic illness population: a systematic review from the Children's Oncology Group. J Pediatr Oncol Nurs.

[14] Landier W (2011). Adherence to oral chemotherapy in childhood acute lymphoblastic leukemia: an evolutionary concept analysis. Oncol Nurs Forum.

[15] Butow P, Palmer S, Pai A, Goodenough B, Luckett T, King M (2010). Review of adherence-related issues in adolescents and young adults with cancer. J Clin Oncol.

[16] (2026). European standards of care for children and adolescents with cancer. https://ccieurope.eu/wp-content/uploads/2025/09/European-Standards-of-Care-for-Children-and-Adolescents-with-Cancer-2025.pdf.

[17] Jones B, Currin-Mcculloch J, Pelletier W, Sardi-Brown V, Brown P, Wiener L (2018). Psychosocial standards of care for children with cancer and their families: a national survey of pediatric oncology social workers. Soc Work Health Care.

